# Comparison of Nanohole-Type and Nanopillar-Type Patterned Metallic Electrodes Incorporated in Organic Solar Cells

**DOI:** 10.1186/s11671-017-2310-7

**Published:** 2017-09-19

**Authors:** Wenyan Wang, Yanxia Cui, Kin Hung Fung, Ye Zhang, Ting Ji, Yuying Hao

**Affiliations:** 1 Key Lab of Advanced Transducers and Intelligent Control System of Ministry of Education, College of Physics and Optoelectronics, Taiyuan University of Technology, Taiyuan, 030024 China; 2 Department of Applied Physics, The Hong Kong Polytechnic University, Hung Hom, Hong Kong

**Keywords:** Surface plasmons, Absorption, Organic solar cells, Resonance, Gratings

## Abstract

Both the nanohole- and nanopillar-type patterned metallic electrodes (PMEs) have been introduced in organic solar cells (OSCs) for improving device performances experimentally, but there is few work addressing the similarities and differences between them. In this theoretical work, we systematically compare the impact of the nanohole- and nanopillar-type PMEs on the performance of an OSC based on hybridized cavity resonances. By optimizing the geometrical parameters of each PME, we obtained an interesting result that the integrated absorption efficiencies in the active layer with different optimized PMEs are almost the same (both are equal to 82.4%), outperforming that of the planar control by 9.9%. Though the absorption enhancement spectra of the two different optimal devices are similar as well, the mechanisms of light trapping at the corresponding enhancement peaks are distinct from each other. In a comprehensive view, the nanopillar-type PME is suggested to be applied in the present system, since its optimal design has a moderate filling ratio, which is much easier to fabricate than its counterpart. This work could contribute to the development of high-efficiency OSCs.

## Background

Manipulation of light by subwavelength metallic nanostructures [1] is an effective way to harvest solar energy into organic solar cells (OSCs) with thin active layers [2–5]. Besides doping chemically synthesized metallic nanoparticles into OSCs [3, 5], it is also very popular to directly pattern the metallic electrode with some subwavelength patterns, i.e., to form a patterned metallic electrode (PME) [6]. It has been reported that PMEs not only can enhance the optical absorption in active layers based on the excitation of the hybridization of plasmonic and photonic modes [7–10] but also can bring forward positive electrical and morphological effects [11–15], resulting in an overall significantly improved performance of thin film photovoltaic devices.

PMEs with one-dimensionally arrayed patterns [8, 9, 14–19] (i.e., 2D PMEs) can be easily fabricated based on the two-beam interference technique [20]; however, the absorption enhancement in OSCs is sensitive to polarization since plasmonic modes cannot be excited at the traverse electric (TE) polarized incidence [10]. PMEs with two-dimensionally (2D) arrayed patterns (i.e., 3D PMEs), which can boost light harvesting efficiency polarization insensitively, have been extensively investigated in the past few years [14, 21–31]. Most of 3D PMEs working as the back contact are opaque. If PMEs are functioned as the front contact, it must be semi-transparent, realized with corrugated thin films [14, 21] or a film with through holes [22, 25]. Except some electrodes with complicated geometries, e.g., the integrated nanopillar-nanowell PME [31], the opaque 3D PMEs are categorized into two types. The first type is to dress the surface of the metallic electrode with some isolated nanoholes [26, 27], which are filled with organic materials in the real OSCs. In other words, the organic materials contacting the PME are in the form of nanopillars. Such kind of PME can be easily obtained by firstly imprinting the active layer with some nanopillars and then thermally evaporating the contact film. By the nanoimprinting technique, Li et al. have demonstrated that a nanohole-type of 3D PME can increase the power conversion efficiency (PCE) by 24.6% with respect to the planar electrode, much superior to the 2D PME [26]. The nanohole-type of PME can also be made from a polystyrene (PS) nanosphere template based on a colloidal self-assembling technique [27]. The other type of opaque 3D PMEs is to decorate some isolated metallic nanopillars on top of a continuous metallic film [23, 24, 28–30], which is exactly the inverse structure of the nanohole one. Le theoretically predicated that there is a great potential of a metallic grating with a 2D array of Ag nanopillars in enhancing the absorption in a thin active layer [24]. We have also theoretically analyzed the influence of the back contact embossed with metallic nanocylinders packed in a hexagonal array on the absorption of a thin OSC device [28]. If the imprinting molds are selected properly, the active layer can be left with some nanoholes, then the following evaporation would make the metal contact protrude into the active layer (i.e., forming the metallic nanopillars) [29, 30]. Zhou et al. showed that the nanopillar PME can increase the PCE of OSC by 9.33% as well as improve the performance of organic light-emitting diodes. Successful applications of the nanopillar-type of PMEs were also witnessed in quantum dot-based solar cells [30]. It is known that nanoholes at a metallic surface excite distinct plasmonic resonances from those of metallic nanopillars loaded on a continuous metal film. Though both types of opaque PMEs were frequently applied in OSCs, there are not enough studies addressing their merit and demerit from a comparison standpoint. Thus, it is of great importance to explore how these two strategies of PMEs work differently from each other in OSCs and which one works better for trapping light into the active layer in theory.

In this work, we constructed models to simulate the two different PMEs applied in a poly[(4,4′-bis(2-ethylhexyl)dithieno[3,2-b:2′,3′-d]silole)-2,6-diyl-alt-(2,1,3-benzothiadiazole)-4,7-diyl] (PSBTBT) and [6,6]-phenyl-C71-butyric acid methyl ester (PC_71_BM) based OSC. The device with nanoholes in the metal electrode is called as Device A and that with metallic nanopillar-type PME is termed as Device B. According to our systematical optimization, it is found that both types of PMEs can produce a 9.9% absorption enhancement in the active layer with respect to the planar electrode, due to the excitation of the hybridization of plasmonic and photonic modes. However, the optimal geometrical parameters of them are completely different and their mechanisms of absorption enhancement are also distinct from each other. Our work provides useful guidance for practical application of PMEs and also contributes to the development of high-efficiency OSCs.

## Methods

**Fig. 1:**
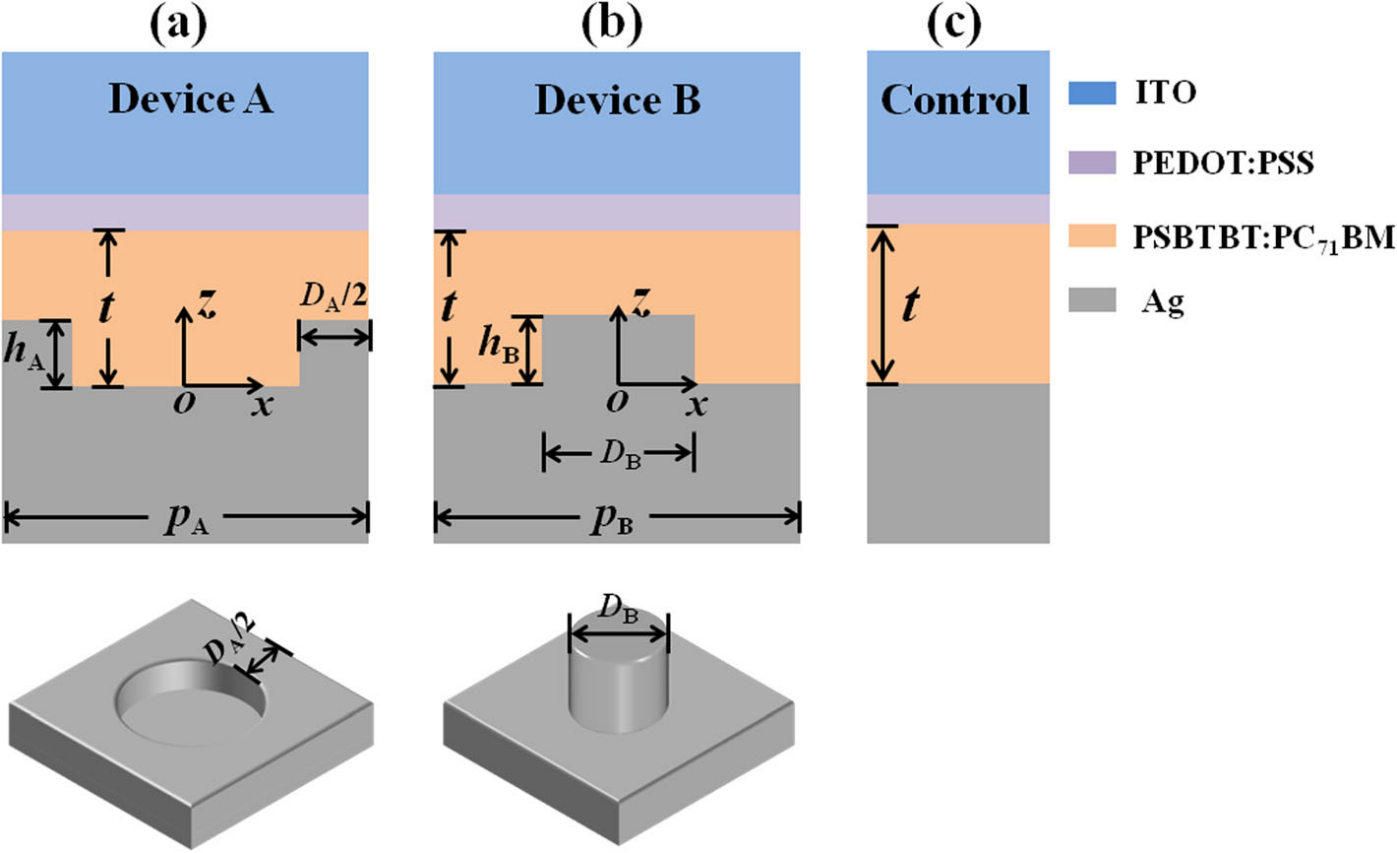
2D diagrams of the OSCs with nanohole-type PME (**a**) and nanopillar-type PME (**b**) as well as the control (**c**). At the cross section, both PMEs have a protruded metal region with a width of *D*, height of *h*, and periodicity of *p*. The subscripts of A and B represent devices with nanohole-type and nanopillar-type PMEs, respectively. The 3D diagram of the nanohole-/nanopillar-type PME is shown below the corresponding device

Figure 1 shows the configurations of OSCs with different PME profiles (Device A and Device B) and the control with planar metallic electrode. The 3D PME diagrams are also included below the corresponding devices for clarity. For simplicity, we consider the isolated nanoholes/nanopillars arranged in square lattice. It is defined that, at the cross-sectional view, PMEs have a protruded metal region with a width of *D*_A_ (or *D*_B_) and a height *h*_A_ (or *h*_B_) in Device A (or Device B). *p*_A_ (or *p*_B_) is the periodicity of the arrayed pattern in Device A (or Device B), and the filling ratio *f*_A_ (*f*_B_) of the protruded metal at the cross-sectional planes is defined as *D*_A_/*p*_A_ (or *D*_B_/*p*_B_). The architecture of the investigated OSCs are ITO/ PEDOT:PSS/PSBTBT:PC_71_BM/Ag. The top ITO layer as the transparent conductive anode has a thickness of 100 nm. The adjacent planar PEDOT:PSS, as the hole transport layer, is 20 nm thick. The active layer is made of PSBTBT:PC_71_BM instead of P3HT:PCBM or PTB7:PCBM because it can absorb more solar energy due to its wide absorption wavelength range (from 350 to 900 nm). Moreover, the calculated results using PSBTBT:PC_71_BM as the active blend can clearly show the potential of absorption enhancement induced by PMEs at long wavelength range when other active blends bear absorption cutoff. The active layer has a thickness of *t*, and its bottom surface follows the pattern of the PME. During the optimization of PMEs, *t* is fixed to 85 nm, the planar control device of the same active layer thickness produces the first absorption peak due to the Fabry–Pérot (FP) cavity resonance. The cathode is made of Ag because it can excite stronger plasmonic modes with comparison of aluminum and copper. In addition, using Ag PMEs, the wavelength range of excited plasmonic modes is broader than that using PMEs made of gold. A thin electron extraction layer which usually locates in-between the active layer and the cathode film is neglected in the optical simulation.

The proposed OSCs are investigated theoretically by the Finite Difference Time Domain (FDTD) method, which has been verified by repeating the work in [32]. All simulations are carried out with periodic boundary conditions applied along both the *x*-axis and *y*-axis and perfectly matched layer (PML) boundaries applied to the top and bottom surfaces. Light are illuminated from the top ITO side at TM (or TE) polarization, which has the electric component along the *x*-axis (or *y*-axis). The wavelength-dependent refractive indices (*n*) of PSBTBT:PC_71_BM are obtained from [33]. And other refractive indices of the materials used in this work are extracted from [18] and [19]. The absorption efficiency of the active layer (*η*) and integrated absorption efficiency (*η*_I_) (over the wavelength range between 350 and 850 nm weighted by the AM1.5G spectrum) are calculated.

## Results and Discussion

**Fig. 2:**
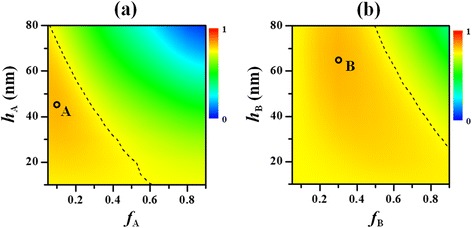
Maps of the integrated absorption efficiency in the active layer (*η*_I_) versus the filling and the height of the arrayed patterns in Device A (**a**) and Device B (**b**) when *p*_A_ (or *p*_B_) = 350 nm. At the denoted point A (with *f*_A_ = 0.1 and *h*_A_ = 45 nm) and point B (with *f*_B_ = 0.3 and *h*_B_ = 65 nm), Device A and Device B, respectively, produce the optimal *η*_I_. The dashed curve represents the contour line of the integrated absorption efficiency equal to that of the planar control

Figure 2a, b shows the maps of *η*_I_ with varied grating height and filling ratio under normal incidence for Device A and Device B, respectively. Here, the periodicities of the PME patterns are fixed as 350 nm, which is an optimized value as shown in Fig. 5c, d. It is observed that the performance of either device depends on both *h* and *f*. For Device A, a shallow metal ridge with a small filling ratio is preferred, while for Device B, a high metal ridge with a moderate filling ratio produces the optimized performance. In detail, the optimized *η*_I_ is achieved at *h*_A_ = 45 nm and *f*_A_ = 0.1 for Device A (i.e., the point A, as denoted in Fig. 2a) and *h*_B_ = 65 nm and *f*_B_ = 0.3 for Device B (i.e., the point B, as denoted in Fig. 2b). It is interesting to find that the optimized *η*_I_ for the two different devices are the same (both equal to 82.4%), enhanced by 9.9% with respect to that of the control (75.0%), though less active material are used in Device A (or Device B). It is noticed the relatively low filling ratio of the optimized Device A, corresponding to a grating ridge with 35 nm width, results in high fabrication difficulties, while the optimized Device B with a filling ratio of 0.3 (i.e., *D*_B_ = 105 nm) can be easily processed through the nanoimprinting techniques [17, 29]. In Fig. 2a, b, the contour line of the integrated efficiency equal to that of the planar control (75.0%) is also indicated by the dashed curve for comparison. Below the dashed curve, *η*_I_ is greater than that of the control and vice versa. Here, it is seen that the region with improved *η*_I_ in Fig. 2b is quite larger than that in Fig. 2a, reflecting that Device B is less sensitive to the geometrical parameters than Device A, which is another merit of the nanopillar-type PME.

It is also noticed that the grating in the optimized Device A is a bit shallower than that in the optimized Device B. It is well known that with the increase of the grating height, the plasmonic modes could get stronger. However, it also brings forward the decrease in the volume of the active material. The combination of these two factors results in an optimal grating height when the *η*_I_ is maximized. However, because the cross-sectional area of the metal protrudes in the *xy* plane for the optimized Device A is around four times greater than that for the optimized Device B, increasing the grating height by the same measure could cause a much greater reduction in the volume of the active material in Device A than in Device B. This might be the reason that the optimal height for Device A is smaller than that for Device B. Our calculation also shows that when the grating height of the optimized Device A increases to 65 nm, the absorption at the short wavelength range (< 600 nm) decays obviously (not shown) due to the apparent reduction in the volume of the active material, whereas, for Device B, decreasing *h*_B_ from 65 to 45 nm yields negligible degradation in absorption over the investigated wavelength range because the change of the volume of the active material is very small.

**Fig. 3:**
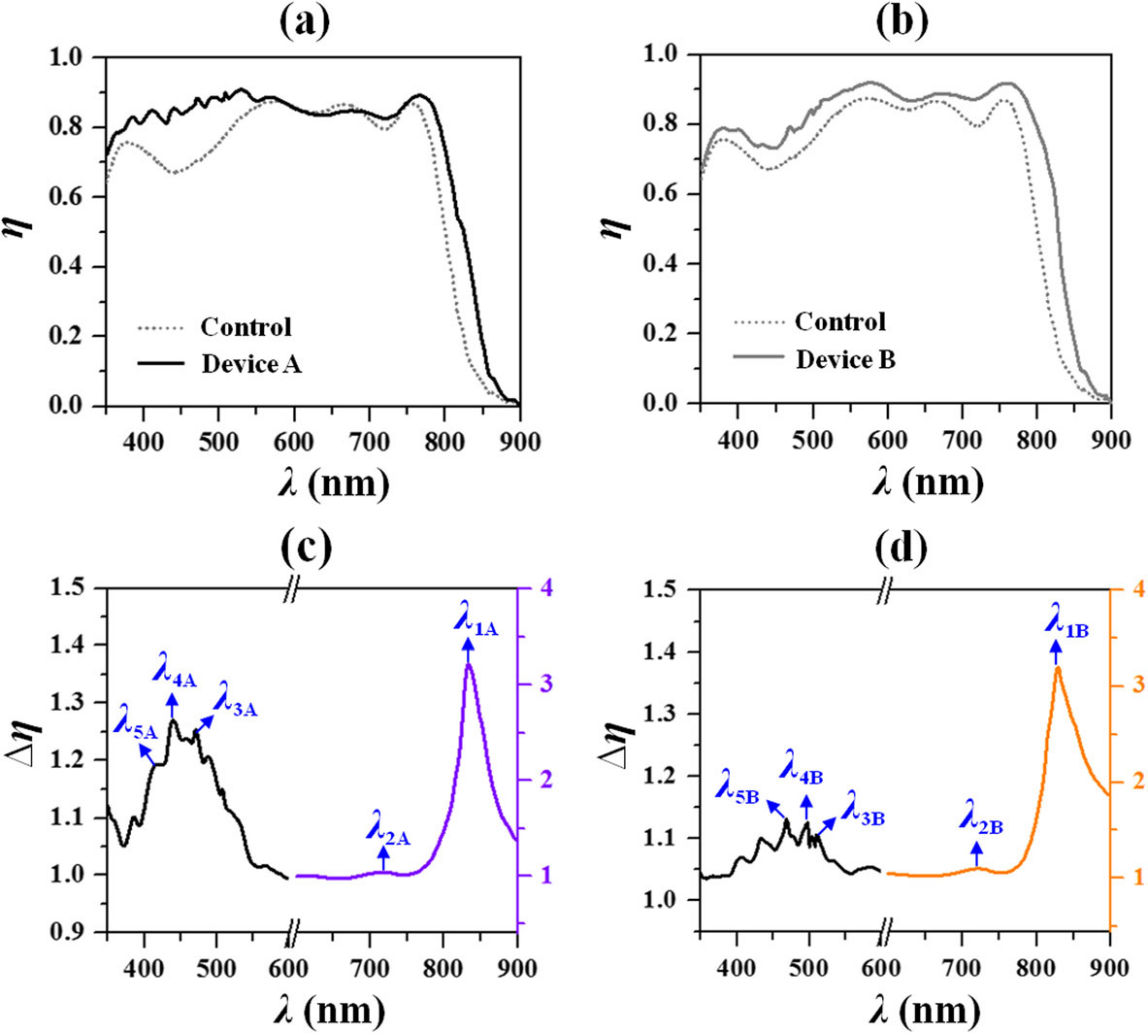
**a** Absorption spectra in the active layer (solid) for Device A (**a**) and Device B (**b**) with respect to that of the planar control (dashed). Spectra of relative absorption change for Device A (**c**) and Device B (**d**). Five enhancement peaks are labeled in **c** with *λ*_1A_ = 830 nm, *λ*_2A_ = 724 nm, *λ*_3A_ = 470 nm, *λ*_4A_ = 440 nm, and *λ*_5A_ = 416 nm, and the other five are labeled in **d** with *λ*_1B_ = 832 nm, *λ*_2B_ = 720 nm, *λ*_3B_ = 510 nm, *λ*_4B_ = 498 nm, and *λ*_5B_ = 468 nm. Device A and Device B are the devices yielding the optimal *η*_I_ in Fig. 2

Figure 3a, b shows the absorption spectra of the optimal Device A and Device B, respectively. For comparison, the absorption spectrum of the control device is also plotted by the dotted line. It is seen in Fig. 3b that the absorption efficiency (*η*) of Device B is greater than that of the control over the whole wavelength range. But for Device A as shown in Fig. 3a, there is a decrease in absorption at the wavelength range around 650 nm; the reason of the integrated absorption efficiency being as high as that of Device B is due to the relatively greater absorption at the wavelength range shorter than 550 nm. To elucidate the physical origins of the observed absorption enhancement, we calculate the relative absorption change for the two optimized devices over that of the control device (∆*η*) (*η*/*η*_control_ − 1) at the investigated wavelength range as shown in Fig. 3c, d. Again, the spectra of the absorption enhancement factor for the two optimized devices display similarities with each other.

**Fig. 4:**
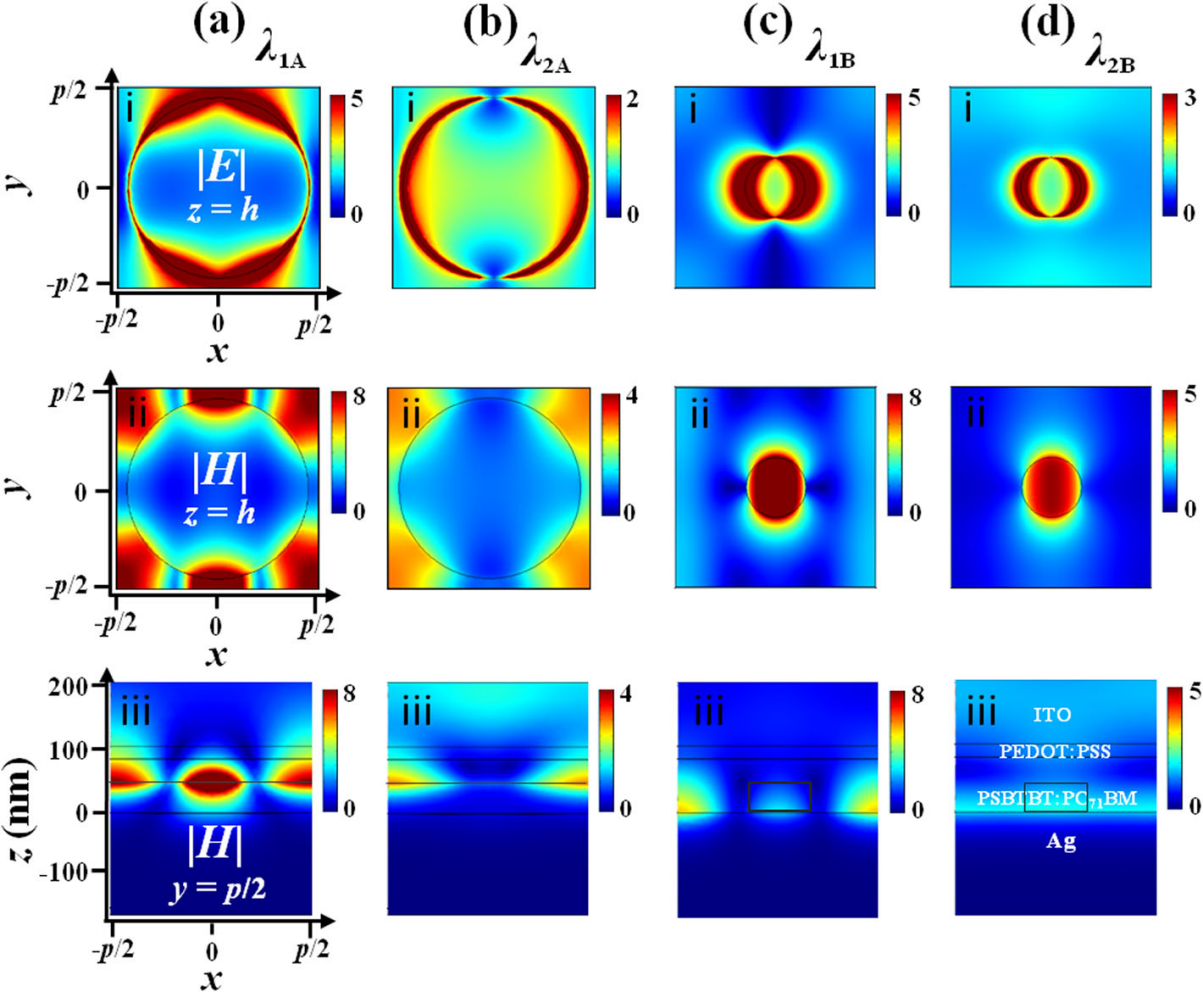
Field maps under TM polarization at different cross sections at the peaks of *λ*_1A_ (**a**), *λ*_2A_ (**b**), *λ*_1B_ (**c**), and *λ*_2B_ (**d**). First row |*E*| at *z* = *h*_A_ or *h*_B_, middle row |*H*| at *z* = *h*_A_ or *h*_B_, and bottom row |*H*| at *y* = *p*_A_/2 or *p*_B_/2. The peaks are as labeled in Fig. 3

At the absorption band edge of the active material, there is an apparent enhancement peak with ∆*η* much greater than 1 [i.e., *λ*_1A_ = 832 nm (or *λ*_1B_ = 830 nm) with ∆*η* = 222% (or 219%) as labeled]. When the wavelength becomes shorter, there is another minor enhancement peak [i.e., *λ*_2A_ = 720 nm (or *λ*_2B_ = 724 nm) with ∆*η* = 4% (or 10%) as labeled]. Figure 4a, b shows the maps of electric and magnetic distributions (under TM polarization) at different cross sections at *λ*_1A_ and *λ*_2A_, respectively. From the maps of |*E*| at *z* = *h*_A_ (subplots of i in Fig. 4a, b), it is seen apparently that the dipole-like localized plasmon resonances (LPRs) are excited along the *y*-axis at *λ*_1A_ and along the *x*-axis at *λ*_2A_, respectively. Although the incident polarization is along the *x*-axis, we witness that the dipole-like LPR at *λ*_1A_ is polarized along the *y*-axis because such a 3D structure can scatter the electric field toward the *y*-axis. From the maps of |*H*| at *y* = *p*_A_/2 (subplots of iii in Fig. 4a, b), we see that propagating surface plasmon polaritons (SPPs) are excited at the metal/dielectric interface at the plane of *z* = *h*_A_, being trapped on top of the metallic protruded ridge due to the reflection from the boundary of nanoholes. However, the trapped modes of |*H*| resonances at these two peaks are of different orders. It is seen that at *λ*_1A_, the |*H*| field at *z* = *h*_A_ (subplot of ii in Fig. 4a) has two nodes (with the minimum amplitude) along the *x*-axis and one node along the *y*-axis, while at *λ*_2A_, there is only one node along both the *x-* and *y*-axes (subplot of ii in Fig. 4b). Influenced from the propagating SPPs, |*E*| at *λ*_1A_ exhibits splitting around the nanohole edge at *x* = 0, which is distorted from the standard dipole-like profile. It is noted at *λ*_2A_, |*E*| inside the nanohole is quite strong because the excitation of propagating SPPs at the metal/dielectric interface at the plane of *z* = 0 (i.e., the bottom of the nanohole) brings forward a constructive interference pattern of |*E*| in the active layer (not shown). For Device B, the maps of electric and magnetic distributions under TM polarization at different cross sections at *λ*_1B_ and *λ*_2B_ are also displayed in Fig. 4c, d, respectively. It is seen from the |*E*| maps at *z* = *h*_B_ that (subplots of i in Fig. 4c, d), for either *λ*_1B_ or *λ*_2B_, the dipole-like LPR is excited along the *x*-axis, but there is an additional bright spot centered at (*x* = 0, *y* = ± *p*_B_/2) taking place at *λ*_2B_. The reason of the generation of this additional bright spot of |*E*| at *λ*_2B_ is similar to that of the strong |*E*| inside the nanohole at *λ*_2A_. Here, the propagating SPPs excited at the bottom of the nanopillar (at the plane of *z* = 0) can be witnessed in the |*H*| map at *y* = *p*_B_/2 (subplots of iii in Fig. 4c, d), resulting in an interference node of |*H*| with minimum amplitude (i.e., a constructive interference region of |*E*|) a certain distance away from the bottom of the nanohole. The constructive interference pattern of |*E*| displays as a bright spot when observed at the planes of *z* = *h*_B_ and of *z* = ± *p*_B_/2 (not shown) at the peak of *λ*_2B_. Differently, at *λ*_1B_, the propagating SPPs are strongly trapped at the plane of *z* = 0 with two nodes formed along the *x*-axis (as shown in the |*H*| map at *y* = *p*_B_/2 in Fig. 4c), which is strongly coupled with the propagating SPPs excited at the top surface of metallic nanopillar (as shown in the |*H*| map at *z* = *h*_B_) (subplots of ii in Fig. 4c, d). Though the propagating SPPs is also excited at the top surface of metallic nanopillar at *λ*_2B_, its amplitude is much lower with respect to that at *λ*_1B_ at the plane of *z* = 0. To sum up, at the afore-investigated two peaks for Device A and two peaks for Device B, the hybridization between the dipole-like LPRs and propagating SPPs is responsible for the trapping of light into the OSC devices.

**Fig. 5:**
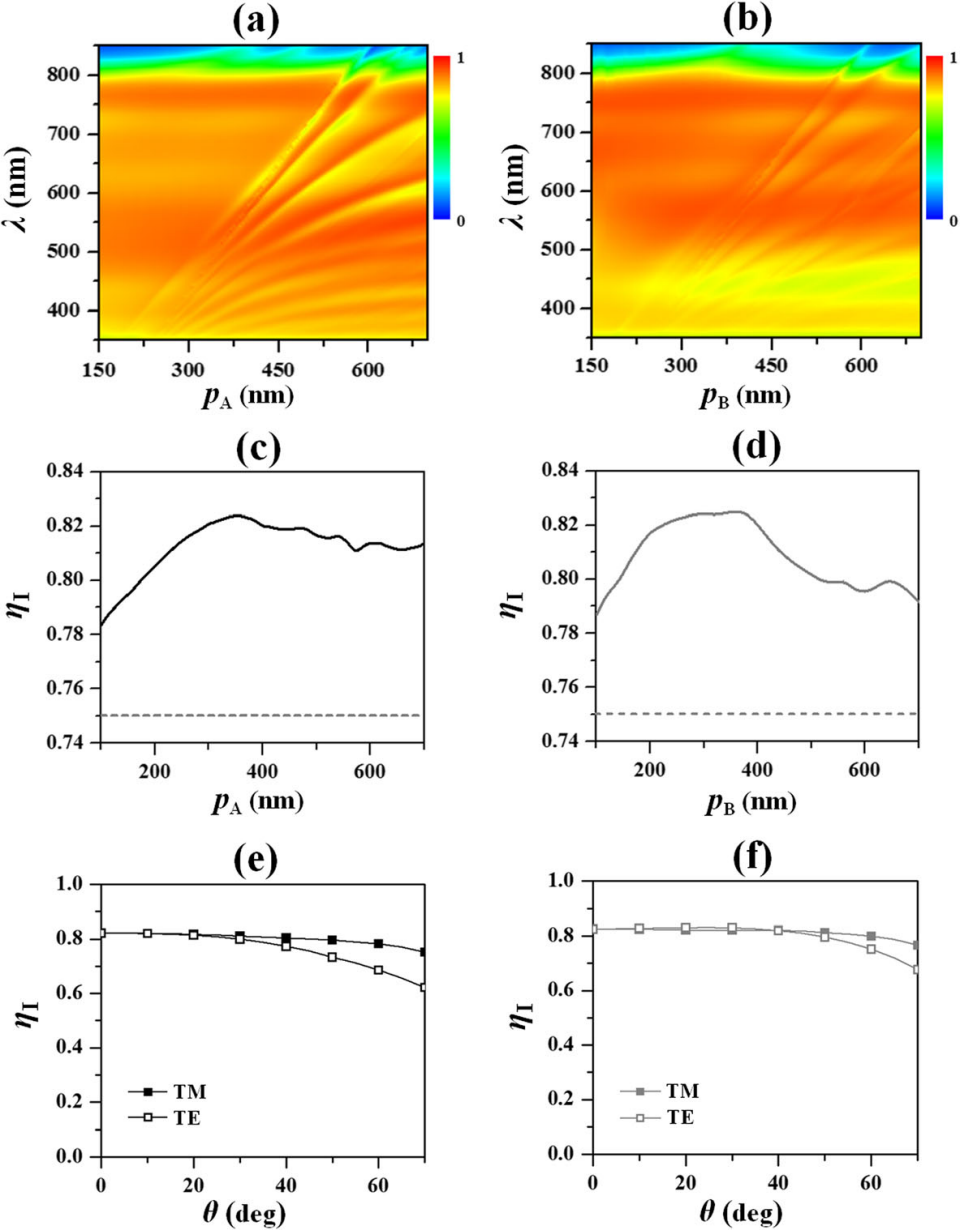
**a**, **b** The absorption spectra when the periodicities of the PME patterns are tuned at normal incidence for Device A (**a**) and Device B (**b**). The integrated absorption efficiency in the active layer (*η*_I_) versus the periodicity for Device A (**c**) and Device B (**d**) with the dashed line representing *η*_I_ for the control device. *η*_I_ versus the incident angle *θ* at TM or TE polarization for the optimal Device A (**e**) and Device B (**f**)

From the enhancement spectra as shown in Fig. 3c, d, one sees that, at the wavelength range shorter than 600 nm, there is a broad enhancement bump with multiple peaks taking place. If the periodicity of the PME pattern decreases, the multiple peaks disappear while only the broad enhancement bump remains. Thus, before looking into the field distributions at the absorption peaks at the short wavelength range, the influences of the periodicity of the PME pattern (*p*_A_ or *p*_B_) on the absorption performance are carried out, with the grating height and filling ratio of the PME for Device A (or Device B) are the same as the corresponding optimal design. Figure 5a, b shows the absorption spectra at tuned periodicities for Device A and Device B, respectively. It is found that, for each device, multiple straight absorption bands which are insensitive to the grating momentum are produced due to localized resonant modes (e.g., the hybridization between dipole-like LPRs and propagating SPPs as presented in this paper). That is exactly the origin of the broad enhancement peak observed at wavelengths shorter than 600 nm. At the same time, there are also some bent absorption bands which are sensitive to the grating momentum formed especially when the periodicity becomes large. It stands to reason that these bent absorption bands are generated due to the phase matching between the propagating constants of SPP modes and reciprocal vectors of the 2D grating (here, there is no in-plane momentum of incident photons at normal incidence). The longer the incident wavelength, the smaller the propagating constant of a certain SPP mode, correspondingly the greater the grating period in order to produce a smaller reciprocal vector for phase matching. When the bent absorption bands cross the straight bands, mode splitting happens, causing the broad enhancement bump with multiple peaks. The integrated absorption efficiency is optimal at *p*_A_ (or *p*_B_) = 350 nm when the localized resonant modes are hybridized with the bent surface modes only over the short wavelength range for Device A (or Device B) as shown in Fig. 5c (or Fig. 5d). At off-normal incidences, the surface modes shift with the incident angle to fulfill the phase matching condition (not shown), even though our study reflects that the integrated absorption efficiencies under either TM or TE polarization are almost angle-insensitive for both devices as shown in Fig. 5e, f.

**Fig. 6:**
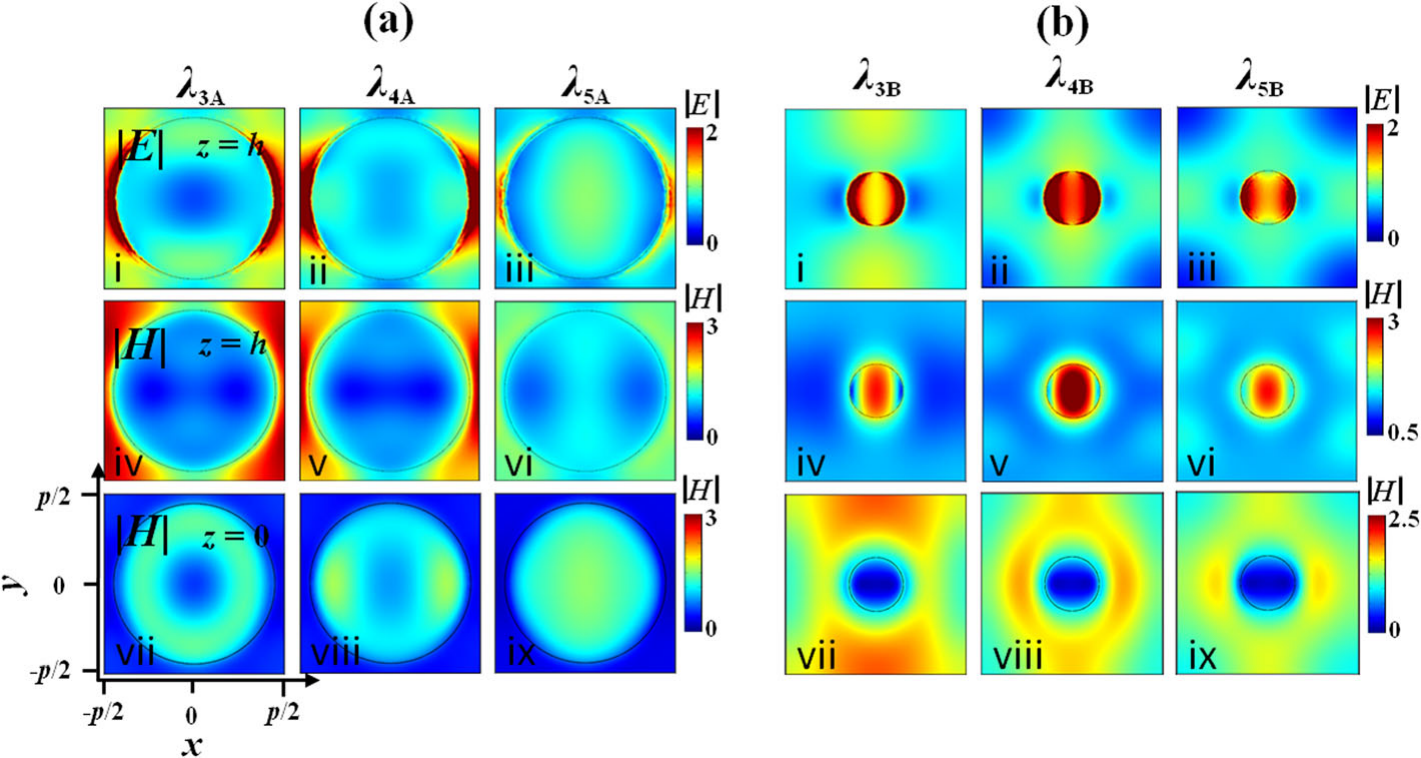
Field maps under TM polarization at different cross sections at the peaks of *λ*_3A_, *λ*_4A_, and *λ*_5A_ (**a**) and *λ*_3B_, *λ*_4B_, and *λ*_5B_ (**b**). First row |*E*| at *z* = *h*_A_ or *h*_B_, middle row |*H*| at *z* = *h*_A_ or *h*_B_, and bottom row |*H*| at *z* = 0. The peaks are as labeled in Fig. 3

Here, we investigate the field distributions of three selected enhancement peaks at the short wavelength range for each device, i.e., *λ*_3A_ = 470 nm, *λ*_4A_ = 440 nm, and *λ*_5A_ = 416 nm as labeled in Fig. 3c and *λ*_3B_ = 510 nm, *λ*_4B_ = 498 nm, and *λ*_5B_ = 468 nm as labeled in Fig. 3d. Figure 6a displays the field maps (under TM polarization) at different cross sections at the three peaks for the optimal Device A. It is seen that the similarities of the maps at different peaks lie on the dipole-like LPRs (as shown from the |*E*| maps at *z* = *h*_A_) (subplots of i–iii in Fig. 6a) as well as the propagating SPPs trapped at the surface of the metallic protruded ridge (as seen from the |*H*| maps at *z* = *h*_A_) (subplots of iv–vi in Fig. 6a). Here, we see that the propagating SPPs at the surface of the metallic ridge has only one node along the *x*-axis but no node along the *y*-axis at *λ*_3A_, *λ*_4A_, and *λ*_5A_, which are different from the cases at *λ*_1A_ and *λ*_2A_. The differences among the resonances at *λ*_3A_, *λ*_4A_, and *λ*_5A_ can be clearly found in the |*H*| maps at *z* = 0 (subplots of vii–ix in Fig. 6a). The envelope of the propagating SPPs at the bottom of the nanohole (*z* = 0) seems like a ring at *λ*_3A_, while an elliptical bar with its long axis directed along the *y*-axis at *λ*_5A_ and a ring plus two elliptical bars with the long axes along the *y*-axis at *λ*_4A_. Figure 6b displays the field maps (under TM polarization) at different cross sections at *λ*_3B_, *λ*_4B_, and *λ*_5B_ for the optimal Device B. At all peaks, the dipole-like LPRs are excited at the top surface of the metallic nanopillar as shown in the |*E*| maps at *z* = *h*_B_ (subplots of i–iii in Fig. 6b). In addition, the propagating SPPs at the top surface of the metallic nanopillars (as shown in the |*H*| maps at *z* = *h*_B_) (subplots of iv–vi in Fig. 6b) are similar at *λ*_3B_, *λ*_4B_, and *λ*_5B_. Besides a bright spot inside the nanopillar, there is also a bright ring produced at the boundary of the nanopillar at *λ*_3B_, *λ*_4B_, and *λ*_5B_, which are different from the cases at *λ*_1B_ and *λ*_2B_. Similar to Device A, the differences among the peaks of *λ*_3B_, *λ*_4B_, and *λ*_5B_ for Device B also lie on the envelopes of the propagating SPPs at the metal/dielectric interface at the plane of *z* = 0 (subplots of vii–ix in Fig. 6b). For both devices, it is the excitations of diverse propagating SPP modes at the bottom of PMEs that cause the broad enhancement bump at the short wavelength range superimposed with multiple tiny peaks.

## Conclusions

In conclusion, the organic solar cells based on nanohole-type and nanopillar-type patterned metallic electrodes have been investigated systematically by comparing their similarities and differences. It has been demonstrated that both of the patterned metallic electrode-based organic solar cells can outperform the planar control with an enhanced light trapping effect in the active layer if optimal designs are utilized. The integrated absorption efficiencies over the investigated wavelength range for the two optimal patterned metallic electrode-based organic solar cells are approximately the same (82.4%), leading to a 9.9% enhancement factor compared to that of the control. Given that the thickness of the active layer in the organic solar cell with either type of patterned metallic electrode is the same as that of the control (which produces the first absorption peak due to cavity resonance), the organic solar cells with patterned metallic electrodes can maintain the carrier transport properties of the planar control device but with enhanced absorption and less active materials. The improved light trapping effects for the two different organic solar cells have also been clarified by analyzing the field distributions at the enhancement peaks. The nanohole-type patterned metallic electrode can excite the dipole-like localized plasmon resonances and propagating surface plasmon polaritons which are localized at the top of metallic ridges. The nanopillar-type patterned metallic electrode can also excite the dipole-like localized plasmon resonances and propagating surface plasmon polaritons which are localized at the top of metallic nanopillars. In addition, grating-coupled surface plasmon polariton modes at the bottom of patterned metallic electrodes are also excited, yielding multiple peaks superimposed over the broad enhancement bump at the wavelength range shorter than 600 nm. The integrated absorption efficiency is optimized with the periodicity of 350 nm when the localized resonant modes are hybridized with the bent surface modes only over the short wavelength range. In a comprehensive view, the nanopillar-type patterned metallic electrode is suggested to be applied in the present organic solar cell system, since its optimal design has a moderate filling ratio, which is much easier to process than its counterpart. The proposed study is expected to contribute to the development of high-efficiency organic solar cells.
